# Considerations for Conducting Web-Based Survey Research With People Living With Human Immunodeficiency Virus Using a Community-Based Participatory Approach

**DOI:** 10.2196/jmir.3064

**Published:** 2014-03-13

**Authors:** Kelly K O'Brien, Patricia Solomon, Catherine Worthington, Francisco Ibáñez-Carrasco, Larry Baxter, Stephanie A Nixon, Rosalind Baltzer-Turje, Greg Robinson, Elisse Zack, The HIV, Health And Rehabilitation Survey Catalyst Team

**Affiliations:** ^1^Department of Physical TherapyFaculty of MedicineUniversity of TorontoToronto, ONCanada; ^2^School of Rehabilitation ScienceFaculty of Health SciencesMcMaster UniversityHamilton, ONCanada; ^3^Canadian Working Group on HIV and RehabilitationToronto, ONCanada; ^4^School of Public Health and Social PolicyUniversity of VictoriaVictoria, BCCanada; ^5^Ontario HIV Treatment NetworkToronto, ONCanada; ^6^Dr Peter AIDS FoundationVancouver, BCCanada; ^7^see Acknowledgements

**Keywords:** HIV infections, Internet, self-report, health surveys, questionnaires, community-based participatory research

## Abstract

**Background:**

Web or Internet-based surveys are increasingly popular in health survey research. However, the strengths and challenges of Web-based surveys with people living with human immunodeficiency virus (HIV) are unclear.

**Objective:**

The aim of this article is to describe our experience piloting a cross-sectional, Web-based, self-administered survey with adults living with HIV using a community-based participatory research approach.

**Methods:**

We piloted a Web-based survey that investigated disability and rehabilitation services use with a sample of adults living with HIV in Canada. Community organizations in five provinces emailed invitations to clients, followed by a thank you/reminder one week later. We obtained survey feedback in a structured phone interview with respondents. Participant responses were transcribed verbatim and analyzed using directed content analysis.

**Results:**

Of 30 people living with HIV who accessed the survey link, 24/30 (80%) initiated and 16/30 (53%) completed the survey instrument. A total of 17 respondents participated in post-survey interviews. Participants described the survey instrument as comprehensive, suggesting content validity. The majority (13/17, 76%) felt instruction and item wording were clear and easy to understand, and found the software easy to navigate. Participants felt having a pop-up reminder directing them to missed items would be useful.

**Conclusions:**

Strengths of implementing the Web-based survey included: our community-based participatory approach, ease of software use, ability for respondents to complete the questionnaire on one’s own time at one’s own pace, opportunity to obtain geographic variation, and potential for respondent anonymity. Considerations for future survey implementation included: respondent burden and fatigue, the potentially sensitive nature of HIV Web-based research, data management and storage, challenges verifying informed consent, varying computer skills among respondents, and the burden on community organizations. Overall, results provide considerations for researchers conducting community-based participatory Web-based survey research with people living with HIV.

## Introduction

Web or Internet-based surveys are increasingly popular in health survey research, enabling researchers to obtain a large amount of information in a cost-effective manner [1,2]. Strengths include the ability for individuals to anonymously complete a questionnaire on their own time at their own pace [3,4]. Nevertheless, Web-based surveys are complex to design and administer for a variety of reasons, including issues surrounding informed consent, risk, anonymity, data storage and security, and sampling [1,5,6]. Response rates with Web-based surveys may be lower compared with paper-based questionnaires further highlighting the importance of carefully considering survey design in relation to the target population [7]. Methodological considerations of Web-based survey research have been considered in other chronic illness populations such as cancer [8], cardiovascular disease [9], Parkinson’s disease [10], and diabetes [11]. Issues conducting Web-based surveys have also been described with men who have sex with men [12,13], and in the context of human immunodeficiency virus (HIV) testing and prevention [14-16]. However, the strengths and challenges of Web-based surveys directly related to people living with HIV are unclear [17].

Community-based participatory research is a “collaborative approach to research that equitably involves all partners in the research process and recognizes the unique strengths that each brings” [18]. Community engagement is a principle of community-based participatory research that collaboratively involves community members, organizational representatives, and researchers in varying degrees of partnership [19,20]. Community engagement in survey research can help to ensure questionnaires are contextually relevant, sensitive, and applicable to the population of interest [21]. In particular, engaging community through a participatory research approach can help to establish strategies to address complexities related to neurocognitive health, fatigue, disclosure, and varying socioeconomic status that may exist for people living with HIV. Internet tools have been used to build capacity for conducting community-based participatory research [22], and guidelines and principles exist for community-based participatory HIV research [19,20,23,24]. However, little is known about the role of HIV community members in guiding Web-based survey research.

In this article, we describe our experience piloting a cross-sectional, Web-based survey with people living with HIV in Canada, using a community-based participatory approach. Specifically, we present feedback from participants living with HIV on our survey implementation, and discuss strengths and considerations for conducting Web-based survey research with people living with HIV.

## Methods

### The Context: Assessing Disability and Rehabilitation Services Use

HIV is increasingly considered a chronic illness in developed countries. More individuals are living longer and aging with the health challenges of HIV, comorbidities, and the side effects of treatment [25-28]. As a result, an increasing number of individuals are now aging with a range of health-related challenges known as disability. Disability is defined as symptoms and impairments (eg, fatigue, weakness, pain), difficulties with day-to-day activities (eg, household chores), challenges to social inclusion (eg, ability to work), and uncertainty or worrying about future health [29]. To provide optimal care for people living with HIV, clinicians and researchers need to understand the range and prevalence of health-related challenges (or disability) experienced by this population [29,30]. We broadly define “rehabilitation”’ as any service or provider who seeks to prevent or address disability experienced by people living with HIV [31]. Rehabilitation can help manage forms of disability such as fatigue, pain, cognitive problems, challenges participating in the labor force, and has the potential to improve health and quality of life outcomes for people living with HIV [32]. Researchers have explored disability and rehabilitation service provision among HIV and health providers and found that despite a high prevalence of disability experienced among people living with HIV, few rehabilitation professionals work in HIV care. However, these concepts have not yet been investigated from the HIV perspective nationally [33-35]. Hence, we developed a survey to establish a profile of disability and rehabilitation services use from the perspective of people living with HIV.

**Figure 1:**
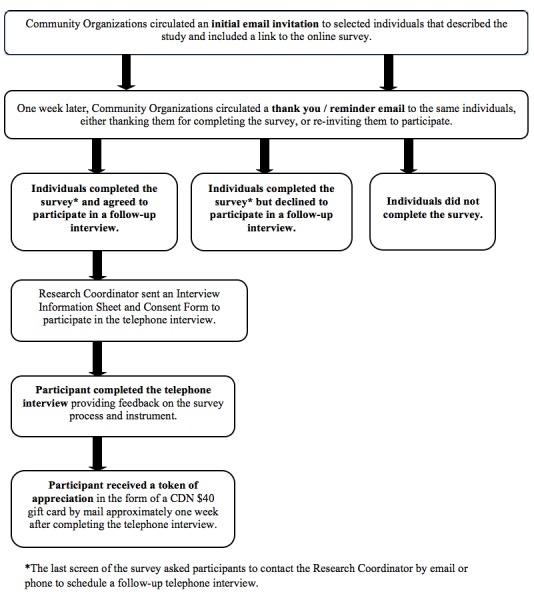
Overview of pilot survey procedure.

We conducted a pilot study of a cross-sectional, electronic survey with a sample of adults living with HIV across Canada using a community-based participatory research approach. Community partner organizations invited individuals to complete the Web-based survey questionnaire followed by a one-on-one structured telephone interview to provide feedback on the survey process (Figure 1).

### HIV Community Partnerships

This research was a community-academic-clinical partnership among academic researchers, people living with HIV, and community-based and health provider organizations. Our team was comprised of eight researchers, five adults living with HIV, one clinician, and seven representatives from community and health provider organizations (categories were not mutually exclusive). Community members provided advice and guidance throughout all phases of this research to ensure successful development and implementation of the survey instrument with the HIV community. The Canadian Working Group on HIV and Rehabilitation (CWGHR) was the principal knowledge user in this research, providing community leadership in all aspects of this work. Our team has a longstanding history of working together in HIV and rehabilitation research. Researchers and community members worked in equal partnership, collectively involved in the conceptualization of the research objectives, application for funding, development of the survey instrument, and implementation and interpretation of the pilot findings. This research was approved by research ethics boards at McMaster University, University of Victoria, and Dalhousie University.

### Survey Instrument Development

Our team of researchers and community members developed a survey instrument called the *HIV, Health and Rehabilitation Survey*, which aimed to describe disability, use of rehabilitation services, and other wellness living strategies used by people living with HIV to manage their health challenges. We developed the instrument collectively as a team, building upon existing frameworks and questionnaires to capture the key constructs of interest. The survey instrument was reviewed, revised, and pre-tested three times by our entire team. Pre-testing involved all team members independently reviewing the questionnaire for content, clarity, and format. Members of the team living with HIV completed the questionnaire as potential participants. We then met for a one-day face-to-face meeting to review the content, wording, format, and administration of the survey instrument and finalize our recruitment process prior to implementation. The final survey instrument was comprised of six components: (1) disability, (2) rehabilitation services use (occupational therapy, physical therapy, speech-language pathology, physiatry, complementary and alternative medicine therapies and providers, AIDS service organizations, and community-based service organizations), (3) comorbidities, (4) living strategies adopted by people living with HIV to deal with HIV and disability, (5) demographic and disease characteristics, and (6) stigma and social support. The survey questionnaire spanned 31 survey screens, four of which were conditional on type of rehabilitation services use. The instrument also included a welcome, eligibility/consent, and instruction page at the beginning and a thank you page at the end. We used LimeSurvey software to administer the survey [36].

### Survey Implementation

We administered the survey instrument electronically via five community organizations using a modified Dillman Tailored Design Method [37] between December 2011 and February 2012. Eligible participants included adults (18 years of age or older) living with HIV in Canada who were able to read and understand English and who had access to the Internet and email. Community organizations (each represented by a community member on our team) circulated an initial email invitation to approximately 7-15 clients who they considered may be eligible for the study. Approximately one week after the initial email was sent out, organizations circulated a thank you/reminder email to the same clients.

As pilot testing aims to gather information from a wide range of potential study participants, recruitment included a combination of formal (email), informal (in-person), and snowball sampling. Sampling was selective, whereby members of organizations specifically approached individuals who they felt would be interested and willing to participate. The primary mechanism of recruitment was email; however, organizations also informally recruited individuals in person at the organization.

### Telephone Interview

At the end of the survey, respondents were invited to take part in a 30-minute structured telephone interview to provide feedback on the survey process and instrument. Interested participants were emailed an information sheet and consent form. During the interview, participants were asked about how well the survey captured their disability, the health services utilized, and the living strategies they used to address the challenges of living with HIV. Participants also were asked about ease of use and readability of the survey instrument and their overall experience with the survey process. Responses were documented verbatim and later analyzed using directed content analysis [38]. Participants were offered a CDN $40 gift card as a token of appreciation for their participation.

## Results

### Recruitment and Participation

At least 56 adults living with HIV were invited to participate in the *HIV, Health and Rehabilitation Survey* pilot, of whom 30 accessed the survey link. Of the 30 who accessed the survey link and introductory page (53% view rate), 24/30 (80%) initiated the survey (participation rate) and 16/30 (53%) completed the survey (completion rate).

Among the 24 adults living with HIV who initiated the survey, 20 (83%) were notified about the survey from one of the five community organizations and 4 (17%) reported that they were forwarded the link from a friend (snowball sampling).

### Demographic Characteristics of Pilot Survey Participants

The median age of survey participants was 52 years (range: 34-63 years) and the majority were men (17/24, 71%) living in a metropolitan geographic area (21/24, 88%). The majority of participants were diagnosed prior to the advent of antiretroviral therapy in the mid-1990s (16/24, 67%), and all were taking antiretroviral medications. Respondents lived in British Columbia, Manitoba, Alberta, Ontario, or Nova Scotia. A total of 22 participants (92%) reported living with at least one concurrent health condition, the most frequent including mental health conditions (14/24, 58%), joint pain (11/24, 46%), muscle pain (10/24, 42%), or addiction (7/24, 29%).

Given our aim was to pilot the *HIV, Health and Rehabilitation Survey*, results focus on the feedback received from participants on the survey instrument and implementation, followed by considerations for conducting community-engaged Web-based survey research with people living with HIV. Results from the full survey implementation will be published in a separate manuscript.

### Pilot Survey Interviews

A total of 17 respondents expressed their willingness to participate in an interview, all of whom provided feedback on the Web-based pilot survey: 16 by telephone interview and one by email. Of these 17 participants, three did not complete the survey questionnaire but followed up with the research coordinator to express his or her interest in participating in the interview. See Table 1 for characteristics of the interview participants.

Table 2 summarizes the perspectives from the pilot participants across six themes: length of time to complete the survey questionnaire, overall thoughts on the Web-based survey, ease of usage and format, software, clarity of questionnaire, and token of appreciation (Table 2).

**Table 1:** Characteristics of interview participants (n=16).

Characteristic		n (%)
**Gender** ^a^		
	Man	12 (70%)
	Woman	2 (12%)
	Other	3 (18%)
Median age (range)		52 years (34-60 years)
**Geographical location (province** ^a^ **)**		
	British Columbia	11 (65%)
	Manitoba	3 (17%)
	Alberta, Ontario, or Nova Scotia	3 (17%)
Median year of HIV diagnosis (range)		1993 (1985-2005)
Number diagnosed prior to the advent of combination antiretroviral therapy (defined as diagnosis before 1996)		10 (59%)
Number taking antiretroviral therapy		16 (100%)
Number born in Canada		14 (88%)
Ever accessed rehabilitation services for HIV or another health condition		10 (62%)

^a^n=17; all other variables out of n=16.

**Table 2:** Participants’ perspectives on the survey instrument and process^a^.

Theme	Pilot survey results
**Length of time to complete the survey questionnaire**	
	Majority of participants (10/15, 67%) completed the Web-based survey questionnaire in approximately 30-45 minutes (range: 10-70 minutes).
	Length of time to complete the questionnaire appeared to be linked with participants’ familiarity with computers.
	Although 7 participants felt 30-45 minutes was an appropriate length of time to complete the survey, overall participants were divided on the appropriate length of time it should take to complete the survey questionnaire.
**Overall thoughts on the Web-based survey questionnaire**	
	Participants described the *HIV, Health and Rehabilitation Survey* questionnaire as “comprehensive”, “detailed”, “straightforward”, which supported content validity in each section.
	Some participants found the instrument “too or very long”, but were unable to suggest items to remove from the instrument, stating all was relevant and important.
	While some participants felt it was burdensome, others wanted more items to further explain their experiences.
**Ease of usage and format**	
	Majority of participants (13/17, 76%) felt instructions and item wording were clear and easy to understand.
	Some participants with English as a second language found challenges with the survey terminology.
	Participants were divided on whether they preferred a questionnaire that could be completed in one sitting versus having a save and return option.
	Some felt if participants were completing the survey in a public space (eg, community organization or library), an anonymous survey that could be completed in one sitting would be essential.
	Others expressed concerns about providing personal information required to save and return later, which could make them less willing to participate in the survey.
**Software**	
	LimeSurvey software was an ideal mechanism in which to administer the survey.
	The majority of participants (13/15, 87%) had no technological difficulties and found the software “straightforward”, “easy to work through, go back and forth”, and “easy to navigate”.
	Some identified potential barriers such as the ability to access a computer and the ability for community organizations to have dedicated computers and space to complete the survey.
	Others raised potential barriers for those not familiar with computers.
	Participants appreciated having no timeout factor, which enabled them to complete the survey at their own pace on their own time. Eight participants used the option to move forward and back during the survey.
	Participants who navigated backwards did so when they realized they had missed certain items, wished to review answers, or wanted to add to an earlier answer when triggered by a later question.
	Participants appreciated having the completion proportion rate (%) at the top of the survey to monitor their progress.
	Eleven participants reported they did not intentionally skip questions in the survey. The majority of participants (12/16, 75%) favored having a pop-up reminder of missed items so that they could choose to go back to complete or confirm that they choose to refuse to answer.
**Clarity of instructions and question wording**	
	Majority of participants (13/16, 81%) felt that the instructions and questions were clear and there was flow to the sequence of items.
	Others found instructions and questions “wordy”, response options “too much” or “a little bit hard to understand”, and after a while reported: “I was just watching the completion bar”.
**Token of appreciation**	
	Participants had varying preferences for the type of token of appreciation for their participation in the research, but felt it should not be less than CDN $20.
	Many liked the choice of an electronic gift card and preferred receiving an honorarium compared with having their name entered in a draw for a larger prize.

^a^Note the denominator may change based on the number of participants who responded to the interview question.

## Discussion

### Overview of Findings

The Web-based *HIV, Health and Rehabilitation Survey* pilot highlighted important considerations for conducting Web-based surveys with people living with HIV. Despite existing reflections on Web-based surveys specific to men who have sex with men [12,13], and in the context of HIV testing and prevention [14-16], to our knowledge, this is the first article to present considerations for implementing a Web-based survey among people living with HIV using a community-based participatory approach. Our aim in conducting this pilot was to evaluate our recruitment and data collection methods, test the software, and refine our survey instrument [39]. Results will directly inform the full implementation of the *HIV, Health and Rehabilitation Survey* with adults living with HIV conducted in partnership with community-based organizations across Canada. Below we provide an overview of the strengths of our approach, and articulate considerations for conducting future community Web-based surveys with people living with HIV. Lessons learned from this pilot study may be more broadly applicable to others conducting community Web-based survey research in other chronic illnesses.

### Strengths of HIV Community Web-Based Survey Research

Strengths of implementing our Web-based self-administered survey included the ease of software use, the ability for participants to complete the questionnaire on their own time at their own pace, and the ability to offer anonymity [3,4]. Well-constructed Web-based surveys may be appealing to respondents, which can increase representativeness of a population. These provide the ability to collect a large amount of data quickly across large geographical areas at a low cost, which is ideal for a national survey that aims to obtain both rural and urban perspectives [3]. Furthermore, evidence suggests response rates and reliability and validity of Web-based health status questionnaires are similar to questionnaires administered with pen and paper, with fewer recruitment efforts among people with chronic disease [40]. Allowing backward and forward navigation and including a pop-up reminder to prompt participants to complete missing responses may help to maximize future survey completion rates.

Our community-based participatory approach to this Web-based survey meant community members and organizations were integrally involved in all aspects of the survey process including development, pre-test, and piloting of the survey instrument. Developing the survey instrument and sampling strategy with community members helped to increase the relevance of the questionnaire to people living with HIV. Community organizations were essential to the recruitment of participants. Team members from the HIV community felt that receiving personal email communications from local community-based organizations may have helped to increase response rates [3]. With community organizations inviting over 50 individuals to participate, we were able to achieve our targeted sample of 30 individuals who accessed the survey link. Although fewer participated in an interview (n=17), we were able to obtain insightful feedback on the survey process and instrument that will enhance the next phase of implementation. Overall, our community-based participatory approach will help to ensure the survey is comprehensive, feasible, and contextually relevant to people living with HIV, while promoting integrated capacity-building and knowledge translation throughout [19].

Although the role of community was initially in an advisory capacity, the nature and extent of engagement increased over the course of this research. Through ongoing team meetings and individual consultations, community members became increasingly experienced and engaged in this work, and invested in the potential impact on their community. This ultimately led to an enhanced partnership and a strengthened and sustainable community-academic research team.

### Considerations for HIV Community Web-Based Survey Research

Our experience illuminated key considerations for conducting Web-based survey research with people living with HIV using a community-based participatory approach.

While it has been suggested that no association exists between survey length and response rate [41], respondent burden and fatigue can increase attrition and missing responses, potentially reducing the validity of HIV Web-based survey research. Computer skills varied among participants resulting in disparities in the length of time to complete the survey and opinions on a feasible timeframe in which to complete the questionnaire. Although no standard length of time is recommended for Web-based surveys [14], researchers are challenged to develop a survey instrument that comprehensively captures the construct of interest while remaining feasible for participants with fewer computer skills in order to maximize response rate and limit sampling bias. Researchers should collaborate with the community to establish the appropriate length of survey instrument for a particular construct.

Completing a Web-based survey may trigger emotional responses, of which the researcher is unaware and unable to offer immediate support [42]. For some participants, completing items about disability and rehabilitation reminded them of the rehabilitation services they had available, evoking feelings of thankfulness, hope, and optimism. Alternatively, completing a questionnaire about disability could evoke feelings of anxiety or uncertainty. Web-based surveys can be useful when asking respondents about sensitive topics that may be difficult to discuss in person [4,5], enabling participants to respond more honestly, and reducing social desirability bias [6,12]. Researchers should consider the sensitive nature of HIV Web-based research and the potential to evoke emotional responses among participants. Removing the term “HIV” in email or e-blast subject headings may help to enhance privacy for those accessing computers in public spaces for whom disclosure is of concern. Providing participants with contact information for the research team and resources to offer support may help to minimize any adverse events [4].

Secure data management and storage to ensure respondent anonymity are essential for Web-based survey research with people living with HIV. Researchers should retain data on a secure server that is inaccessible to other parties and that is approved by their research ethics boards. Information regarding anonymity and data storage should be clearly articulated to participants in the introductory email and survey page so that individuals can make an informed decision about participation. Maintaining anonymity can be challenging when researchers aim to provide a token of appreciation to respondents [12]. Constructing a separate “token of appreciation” survey where respondents are invited to enter an email address to receive an electronic gift card, separate from the survey, may help to ensure anonymity of survey responses, but may still identify respondents as participants in the research. Furthermore, offering incentives in the context of an anonymized survey could lead to respondents duplicating or falsifying survey responses in order to take advantage of study incentives. Further research examining how to provide study incentives that do not encourage multiple survey submissions is needed [13].

Web-based research makes it difficult to verify informed consent. Volunteers independently interpret the purpose, risks, and benefits of participating in the research (often stated in the email invitation or introductory page of the instrument) and consent is implied based on the completion of consent items and the questionnaire. No opportunity exists for dialogue between the researcher and the participant to determine capacity to consent and complete the survey. In our study, we were unable to determine whether respondents truly understood the research and the questions asked, and we were limited in our ability to provide opportunities for clarification [4,12,42]. This has further implications for people living with HIV who may have varying neurocognitive ability. All components of the consent process including declaration of the study purpose, institutions behind the study, risks and benefits, and how privacy will be maintained should be made available online for the respondent [43,44]. Having clear questions that review eligibility criteria and asking participants to confirm their understanding of what is involved in participating may help confirm they have read the information and agree to participate in the study. Providing contact details of the research team also can help participants clarify details of informed consent [5].

Lack of computer and Internet access, and variability in computer skills may pose barriers for people living with HIV who wish to participate in Web-based surveys, resulting in potential selection bias and reduced generalizability [43]. Variation in Internet speed and devices such as desktop, laptop, or tablet, may pose implications for font size and further influence the ability to navigate Web-based surveys [37,45]. While resource intensive, researchers should consider having Internet-accessible computers and research or peer personnel support available within community-based organizations to maximize participation.

Finally, our community-based participatory research approach may be burdensome for community organizations with little time or mandate to engage in research beyond the scope of their current programs and services. Timelines of the email distribution varied depending on the workload across the five community organizations and it was difficult to ascertain the number of people who received the invitation email. Ongoing tailored communication with each organization is important for strategizing ways to maximize their ability to engage in HIV Web-based survey research with minimal burden.

### Limitations

Our study is not without limitations. Respondents primarily included men over 50 years old living with HIV, recruited from AIDS Service Organizations, who were more likely to self-select to participate in the research. Hence, while our completion rate was slightly higher than 40% reported in a meta-analysis of 68 electronic surveys [3], respondents may not be representative of the broader HIV population. Estimates suggest that approximately only one-third of adults living with HIV in Ontario access an AIDS Service Organization [46,47]. These respondents may be linked with AIDS Service Organizations to access social support and services, suggesting they may have increased severity of self-identified health-related challenges compared with the broader HIV population. Moreover, Web-based surveys may not translate into higher response rates compared to other methods such as mailed surveys [48]. Increasing the number of email communications to three, ensuring email communication is personalized, along with pre-notification of the survey may help to increase response rate [3]. Finally, while concerns of multiple responses by the same person in Web-based surveys exist [4,43,49], we did not feel this was an issue in our pilot study, given the personalized nature of recruitment and length of reported time it took to complete the questionnaire. Researchers may consider monitoring IP addresses and assigning a unique identifier to every questionnaire viewer to determine participation rates and filter multiple responses [43]. However, this may not be feasible if surveys are completed by different respondents on the same computer at collaborating organizations.

### Conclusions

Our experience piloting the *HIV, Health and Rehabilitation Survey* highlighted important considerations for the implementation of Web-based surveys with people living with HIV. Strengths included our community-based participatory approach, ease of software use, ability to complete the questionnaire on one’s own time at one’s own pace, opportunity to obtain geographic variation, and ability for anonymity. Considerations include respondent burden and fatigue, the potentially sensitive nature of HIV Web-based research and inability for researchers to provide immediate support, data management and storage, challenges verifying informed consent, varying computer skills among respondents, and the burden on community organizations. Results provide strategies for enhancing community participatory Web-based survey research in the field of HIV.
